# An Engineered Viral Protease Exhibiting Substrate Specificity for a Polyglutamine Stretch Prevents Polyglutamine-Induced Neuronal Cell Death

**DOI:** 10.1371/journal.pone.0022554

**Published:** 2011-07-20

**Authors:** Saravanan Sellamuthu, Bae Hyun Shin, Hye-Eun Han, Sang Min Park, Hye Jin Oh, Seong-Hwan Rho, Yong Jae Lee, Woo Jin Park

**Affiliations:** 1 College of Life Sciences, Gwangju Institute of Science and Technology (GIST), Gwangju, Korea; 2 Physics Institute, University of Freiburg, Freiburg, Germany; 3 Department of Pharmacology, Yale University School of Medicine, New Haven, Connecticut, United States of America; University of Melbourne, Australia

## Abstract

**Background:**

Polyglutamine (polyQ)-induced protein aggregation is the hallmark of a group of neurodegenerative diseases, including Huntington's disease. We hypothesized that a protease that could cleave polyQ stretches would intervene in the initial events leading to pathogenesis in these diseases. To prove this concept, we aimed to generate a protease possessing substrate specificity for polyQ stretches.

**Methodology/Principal Findings:**

Hepatitis A virus (HAV) 3C protease (3CP) was subjected to engineering using a yeast-based method known as the Genetic Assay for Site-specific Proteolysis (GASP). Analysis of the substrate specificity revealed that 3CP can cleave substrates containing glutamine at positions P5, P4, P3, P1, P2′, or P3′, but not substrates containing glutamine at the P2 or P1′ positions. To accommodate glutamine at P2 and P1′, key residues comprising the active sites of the S2 or S1′ pockets were separately randomized and screened. The resulting sets of variants were combined by shuffling and further subjected to two rounds of randomization and screening using a substrate containing glutamines from positions P5 through P3′. One of the selected variants (Var26) reduced the expression level and aggregation of a huntingtin exon1-GFP fusion protein containing a pathogenic polyQ stretch (HttEx1(97Q)-GFP) in the neuroblastoma cell line SH-SY5Y. Var26 also prevented cell death and caspase 3 activation induced by HttEx1(97Q)-GFP. These protective effects of Var26 were proteolytic activity-dependent.

**Conclusions/Significance:**

These data provide a proof-of-concept that proteolytic cleavage of polyQ stretches could be an effective modality for the treatment of polyQ diseases.

## Introduction

The aggregation of polyglutamine (polyQ) proteins within neuronal cells has been implicated in the pathogenesis of a group of neurodegenerative disorders, including Huntington's disease (HD) [1], [2]. The toxicity of aggregates in neurons is attributed to altered proteasomal functions [3], [4] and/or to the sequestration of vital cellular proteins such as transcriptional elements [5], molecular chaperons [6], cytoskeletal proteins [7], and components of the ubiquitin-proteasome system. Therefore, aggregation of polyQ proteins is widely thought of as an attractive therapeutic target.

Various interventions have been shown to be effective in inhibiting polyQ protein aggregation and thereby, in reducing the toxicity of these proteins in cultured cells and animal models. Small peptides, referred to as glutamine binding peptides (QBP) that preferentially bind to pathogenic polyQ stretches were identified by screening a combinatorial peptide library. It was also shown that the expression of QBP tandem repeats in cultured cells inhibits polyQ-induced cell death [8]. In another study, suppressor polypeptides with a flexible helix spacer sequence flanked by two 25Q sequences were found to be effective in Drosophila models of HD [9]. Small chemical compounds isolated from yeast-based high throughput screens potently inhibited polyQ aggregation in brain slice cultures and other cell-based models [10]. A number of chemical compounds, such as Congo Red, Thioflavin S, Chrysamine G, and Direct Fast Yellow, have also been shown to inhibit polyQ aggregation [11]. In addition, there are reports claiming the effective use of intracellular antibodies that specifically bind to elongated polyQ chains in HD models [11], [12], [13,]. Finally, over-expression of molecular chaperons, such as Hsp70 and Hsp40, in cellular and Drosophila models of HD also significantly reduced aggregation and consequentially prevented neurodegeneration [14], [15]. These compounds and molecules may block aggregation by selectively binding to and stabilizing the native conformation of the elongated polyQ tract.

We hypothesized that the proteolytic cleavage of pathogenic polyQ stretches would help reduce the level of aggregation by shortening the pathogenic polyQ stretch to a non-pathogenic length, thereby greatly reducing the complications induced by polyQ proteins. Since no protease is known to cleave polyQ stretches, we decided to adopt a directed evolution approach to generate a polyQ-specific protease. The polyprotein processing of members of the picornaviridae family of viruses, which includes Hepatitis A virus (HAV), is mainly mediated by the 3C proteases (3CP). These proteases show a unique substrate specificity preference for a glutamine residue at the P1 site. We have previously utilized a yeast-based screening method, referred to as the Genetic Assay for Site-specific Proteolysis (GASP), to produce an engineered variant of HAV 3CP that can cleave a peptide substrate containing glutamine at the P1′ site more efficiently than its original substrate [16]. Motivated by this earlier success, we further extended the use of GASP to generate a polyQ-specific protease (PQP) using directed evolution approach. The results of this study clearly validate our screening methodology and our PQP development approach. This study also shows that one of the selected variants (Var26) prevents polyQ protein aggregation and attenuates polyQ-induced cytotoxicity. These data provide a proof-of-concept that proteolytic cleavage of polyQ stretches may be a valuable strategy for the treatment of polyQ diseases.

## Results

### Glutamine scanning of the HAV 3C protease

**Figure 1:**
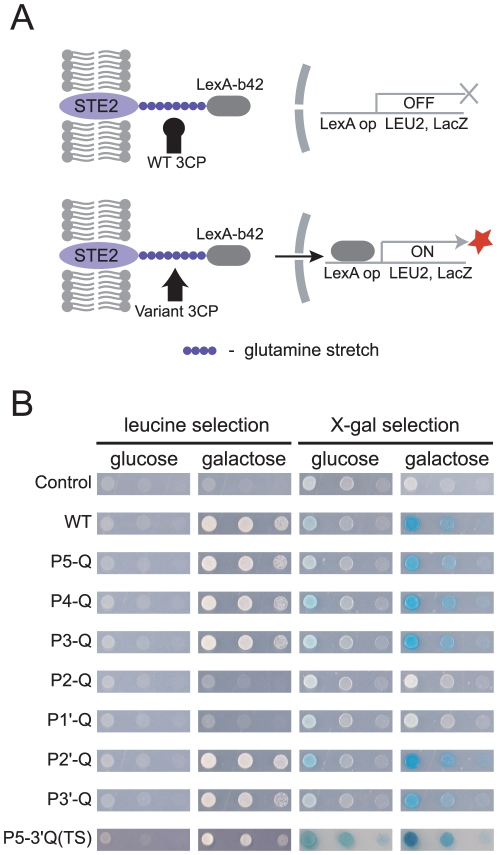
Principle of GASP. **A.** A fusion protein containing the Ste2 transmembrane domain followed by a polyQ substrate linker and a LexA-b42 transcription factor was expressed in yeast EGY48 cells using a constitutive ADH promoter. Co-expression of galactose-inducible WT HAV 3CP does not result in cleavage of the polyQ substrate sequence (upper panel), while a conceptual 3CP variant cleaves the polyQ linker, causing the release of the transcription factor from the membrane, which in turn activates the reporter genes, Leu2 and LacZ (lower panel). **B.** EGY48 cells were transformed with pGAL-HAV3CP and pADH-Ste2-Substrate-Lex in which glutamine is substituted in the P5-P3′ positions of the 3CP substrate sequence (see Table S1). Cleavage of the indicated substrate sequence was evident by the growth of yeast cells in leucine-deficient medium and the formation of a blue color on X-gal medium.

The principle underlying GASP was previously published and is illustrated in Fig. 1A. Cleavage in the substrate site of a membrane-anchored fusion protein (STE2-substrate-LexA-b42) induces the release of the LexA-b42 moiety from the plasma membrane and activates the expression of the reporter genes, *Leu2* and *LacZ*. Protease expression is under the control of the *Gal1* promoter and is, thus induced by the addition of galactoseto the media. Growth on selective plates and formation of a blue color on X-gal plates indicates site-specific proteolysis. Using this method, the ability of the HAV 3C protease (3CP) to cleave substrates containing glutamine substituted at different sites was analyzed. Substrate linker oligonucleotides encoding the wild-type (WT) substrate sequence (ELRTQ↓SFS) and mutated substrate sequences (P5-Q ∼ P3′-Q) in which glutamine residues replaced the native residues from P5 through P3' (Table S1) were prepared and cloned into the pADH-Ste2-Lex vector. Each of these substrate vectors was transformed, along with the protease vector, pGAL-3CP, into the yeast strain EGY48. The results from the selective plates and the X-gal plates suggested that glutamine is tolerated at the P5, P4, P3, P1, P2′, and P3′, but not at the P2 and P1′ positions (Fig. 1B). An oligonucleotide encoding a substrate (P5-3′Q(TS)) containing glutamine residues at all sites except the P2 and P1′ positions (QQQTQ↓SQQ) was then prepared. This substrate was cleaved by 3CP (Fig. 1B). Therefore, the S2 and S1′ pockets were subjected to further engineering to generate PQP.

### Engineering at the S2 pocket

In a prior study, we had engineered the S2 pocket of 3CP and generated a number of variants [16]. From this group, 12 variants were selected that contained mutations at amino acids 145, 146, 147, and 155. This work revealed two key points: first, mutating the 29^th^ amino acid is critical for the glutamine accomodation in the S2 pocket; and second, the amino acid 155 is favored by the original leucine. Therefore, we decided to include M29 and exclude L155 for additional mutation screening. In addition, the V28 residue was also included because it is close to M29 and appears to play a role in constituting the S2 pocket based on the 3CP crystal structure. Since the resulting complexity would be too large if all of the five target residues were randomized at once, we decided to perform randomized mutagenesis of four residues at a time, V28/M29/H145/K146 and M29/H145/K146/K147. These residues were randomized by successive PCR and screened for their proteolytic activity using the P2-Q substrate (ELRQQ↓SFS). From these separate screens, 23 and 19 variants were obtained, respectively (Tables S2 and S3).

### Engineering at the S1′ pocket

A close examination of the 3CP crystal structure suggested that three amino acids (L168/P169/L199) might constitute the S1′ pocket. These residues were randomly mutated by successive PCR. The resulting mutant library was screened for proteolytic activity using the P1′-Q substrate (ELRTQ↓QFS). Twenty positive clones were selected based on colony growth on selective plates and blue color formation on X-gal plates (Table S4). 

### Combining the S2 and S1′ pocket variants

**Figure 2:**
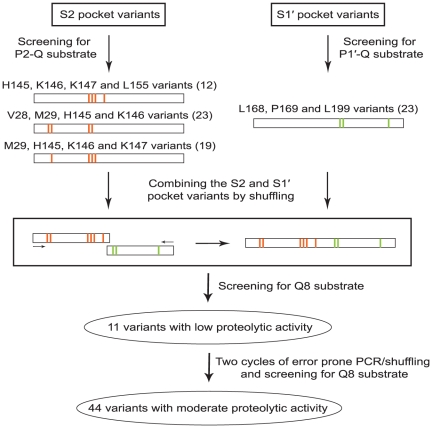
Engineering scheme for the isolation of polyQ cleaving variants. Screening of the S2 pocket variants for P2-Q cleavage yielded 54 positive candidates (12 from HKKL, 23 from VMHK, and 19 from MHKK libraries). Screening of the S1' pocket variants for P1'-Q cleavage resulted in 23 clones from the LPL library. These variants were combined through shuffling to generate a pool of variants in which each protease possesses S2 as well as S1' pocket mutations. These variants were then screened for Q8 substrate cleavage yielding 11 candidates with low activity. These mutants were further subjected to two rounds of error prone PCR and shuffling coupled with selection through GASP to generate 44 variants with moderate activity.

The individually selected variants from S2 pocket and S1′ pocket engineering cleaved the P2-Q and P1′-Q substrates respectively, but none of the selected variants could cleave the polyQ substrate (data not shown). Therefore, the variants from the four independent screenings were combined by shuffling to generate a pool of variants that contained various mutations in both the S2 and S1′ pockets (Fig. 2). The resulting library was screened using the Q8 substrate (QQQQQ↓QQQ), which contains glutamine at all eight sites, and eleven positive clones were selected (Table S5). All the selected variants appeared to have relatively low proteolytic activity, as the colonies grew slowly on selective plates and produced a faint blue color on X-gal plates (data not shown). These eleven candidate clones were pooled together and were used as template for further mutagenesis by error prone PCR and DNA shuffling. After two rounds of mutagenesis and selection, 44 variants that appeared to have moderate proteolytic activity against the Q8 substrate were finally obtained (Table S6). The clone that appeared to have the highest proteolytic activity, variant 26 (Var26), was tested for its effects in an in vitro model of HD.

### Var26 prevents aggregation of polyQ proteins

**Figure 3:**
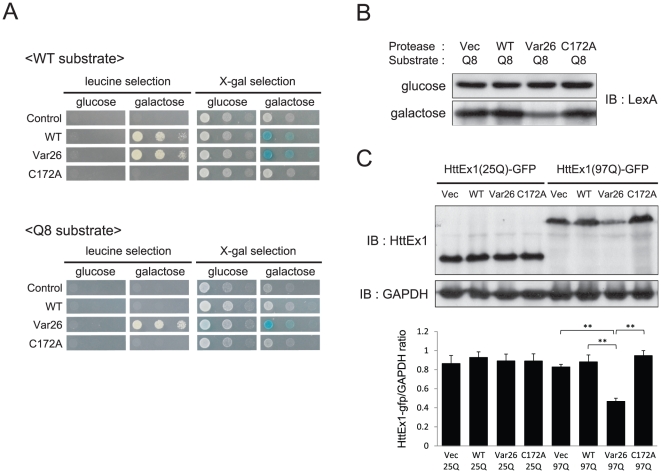
Var26 cleaves polyQ stretches. **A.** Yeast EGY48 cells containing either an empty vector (Vec), pGAL-3CP (WT), pGAL-Var26 (Var26), or pGAL-C172A (C172A) plasmids, together with pADH-Ste2-2B/2C-Lex (WT substrate) or pADH-Ste2-Q8-Lex vectors (Q8 substrate) were plated on leucine-lacking plates and X-gal plates containing either glucose or galactose. **B.** The pADH-Ste2-Q8-Lex vector was co-transformed with an empty vector (Vec), or pGAL-3CP (WT), pGAL-Var26 (Var26), or pGAL-C172A (C172A) plasmids into EGY48 yeast cells and grown in inducible (galactose) or non-inducible (glucose) medium. After 16 hours of incubation, cells were lysed, separated by SDS-PAGE, and blotted and probed with anti-LexA antibody. **C.** SH-SY5Y cells were transfected with pcDNA-HttEx1(25Q) or (97Q)-GFP together with an empty vector (Vec) or, pcDNA3-WT 3CP (WT), pcDNA3-Var26 (Var26), or pcDNA3-C172A (C172A) plasmids. Cell lysates were separated by SDS-PAGE, blotted, and probed with an anti-huntingtin antibody. GAPDH was used as a loading control. The expression ratio of HttEx1 and GAPDH was calculated using computer-assisted image analysis. Error bars represent the SD. Significance was determined by student's *t* test. *p<0.05, **p<0.01.

Both WT 3CP and Var26 cleaved the WT substrate but only Var26 cleaved the Q8 substrate in yeast as assessed by GASP (Fig. 3A). A C172A mutant of Var26 was generated in which the catalytic cystein^172^ nucleophile was mutated to alanine [17], [18], [19], [20]. C172A did not cleave the Q8 substrate, indicating that the cleavage of the Q8 substrate in yeast requires the proteolytic activity of Var26 (Fig. 3A). These data showed that Var26 acquired a relaxed rather than an altered substrate specificity.

Yeast cells were transformed with a Q8 substrate-expression vector along with WT 3CP-, Var26-, or C172A-expressing vectors. Protein extracts were blotted and probed with an anti-LexA antibody to detect the full-length STE2-substrate-LexA-b42 fusion protein. Without protease expression induction (glucose), no change in the level of the fusion protein was detected. When the expression of the protease was induced (galactose), Var26, but not WT 3CP or C172A, significantly reduced the amounts of the full-length fusion protein (Fig. 3B), which confirmed our GASP results.

SH-SY5Y [21], a human neuroblastoma cell line, was then transfected with plasmids encoding a human huntingtin (Htt) exon1-GFP fusion protein containing either a non-pathogenic stretch (25Q) of glutamines (HttEx1(25Q)-GFP) or a pathogenic stretch (97Q) of glutamines (HttEx1(97Q)-GFP) along with the protease expression plasmids. None of the tested proteases affected the expression level of HttEx1(25Q)-GFP, but Var26 significantly reduced the levels of HttEx1(97Q)(Fig. 3C). This result indicates that Var26 effectively cleaves the polyQ stretch in HttEx1(97Q)-GFP in mammalian cells. It was surprising that Var26 did not cleave the polyQ stretch in HttEx1(25Q)-GFP. It may be possible that these two polyQ stretches adopt different conformations.

**Figure 4:**
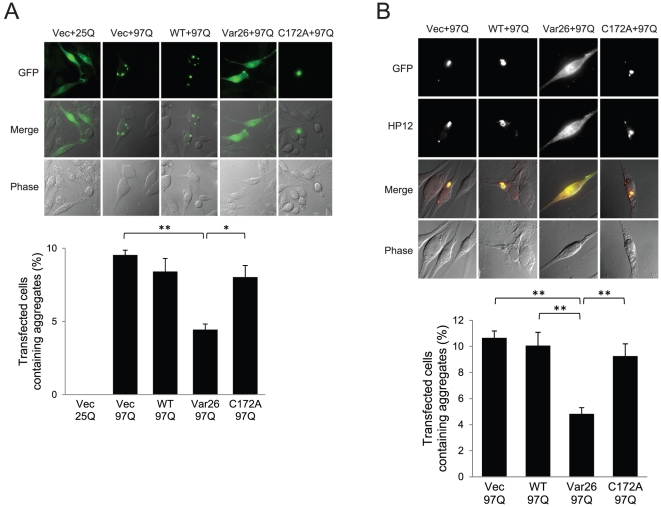
Var26 prevents polyQ aggregation. **A.** SH-SY5Y cells were cotransfected with pcDNA-HttEx1(25Q) or (97Q)-GFP together with an empty vector (Vec) or, pcDNA3-WT 3CP (WT), pcDNA3-Var26 (Var26), or pcDNA3-C172A (C172A). After 48hrs of incubation, the cells were fixed and observed under a fluorescence microscope. The percentage of GFP-aggregation positive cells was plotted (n = 3). Error bars represent the SD. Significance was determined by student's *t* test. *p<0.05, **p<0.01. **B.** SH-SY5Y cells were treated as in panel A. After 48hrs of incubation, the cells were fixed and immunostained with the HP12 antibody. The percentage of cells containing amino-terminal aggregation was plotted (n = 4). Error bars represent the SD. Significance was determined by student's *t* test. **p<0.01.

Aggregation of the HttEx1-GFP fusion proteins was observed under a fluorescent microscope. Consistent with previous reports, no aggregation was observed with HttEx1(25Q)-GFP but significant aggregation was observed with HttEx1(97Q)-GFP. This protein aggregation was significantly blocked by Var26 but not by WT 3CP or C172A (Fig. 4A). This result indicates that cleavage of the polyQ stretch by Var26 leads to the dispersion of the resulting carboxy-terminus of the fusion protein. To test whether the amino-terminal fragment of the fusion protein that results from Var26-mediated cleavage is also dispersed, immunostaining was performed with the antibody HP12, which is specific for the amino-terminal 17 amino acids of Htt. The aggregation of the amino-terminal fragments was also significantly blocked. Moreover, HP12 immunofluorescence was indistinguishable from GFP fluorescence in all of the cells observed (Fig. 4B). This result indicates that the cleavage of HttEx1(97Q)-GFP by Var26 reduced aggregation of the fusion protein.

### Var26 prevents polyQ-mediated cytotoxicity

**Figure 5:**
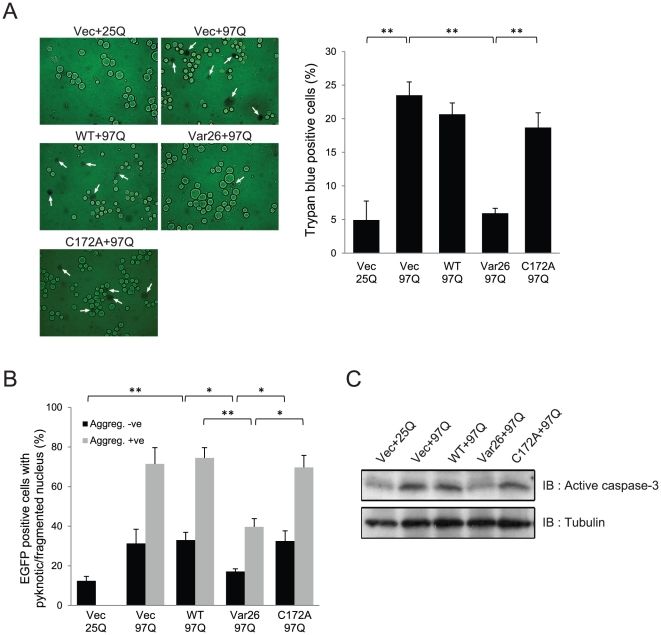
Var26 prevents polyQ-induced cell death. **A.** SH-SY5Y cells were cotransfected with pcDNA-HttEx1(25Q) or (97Q)-GFP together with an empty vector (Vec) or, pcDNA3-WT 3CP (WT), pcDNA3-Var26 (Var26), or pcDNA3-C172A (C172A). After 48 hrs of incubation, the cells were stained with trypan blue and viewed under a microscope. Arrows indicate cells stained with trypan blue. The percentage of dead cells was plotted (n = 3). Error bars represent the SD. Significance was determined by student's *t* test. *p<0.05, **p<0.01. **B.** In a different experiment, the same group of cells was examined for, pyknotic nuclei by Hoechst staining. The number of pyknotic nuclei was measured and is plotted (n = 3). Error bars represent the SD. Significance was determined by student's *t* test. *p<0.05, **p<0.01. **C.** Cells harboring the plasmids described in panel A were lysed, separated by SDS-PAGE, and blotted and probed with a caspase-3 antibody. Tubulin was used as a loading control.

HttEx1(97Q)-GFP induced significant SH-SY5Y cell death as assessed by Trypan blue staining, which was greatly reduced by Var26 but not by WT 3CP or C172A (Fig. 5A). Further, in a separate experiment the expression of WT or Var26 or C172A alone did not significantly affect the viability of the cells (see Fig. S1). Cell death can also be monitored by nucleus fragmentation and condensation, a phenomenon known as pyknosis. Consistent with previous reports [22], HttEx1(97Q)-GFP induced pyknosis in cells both with and without aggregation. Var26 significantly reduced the number of pyknotic cells in the presence or absence of aggregation, but WT 3CP or C172A did not (Fig. 5B). Caspase 3 is an execution caspase mediating apoptosis. HttEx1(97Q)-GFP elevated the level of active caspase-3, which was significantly suppressed by Var26 but not by WT 3CP or C172A (Fig. 5C). These results indicate that Var26 inhibits polyQ-induced cell death in SH-SY5Y cells in a proteolytic activity-dependent manner.

## Discussion

A group of neuronal diseases are caused by the accumulation and/or aggregation of amyloidogenic proteins. For example, a primary cause of Alzheimer's diseases (AD) is thought to be the accumulation of a short peptide called amyloid-β (Aβ) in the brain. Elevated degradation of Aβ by an endogenous protease, such as neprilysin [23], [24], or a viral protease, such as turnip mosaic virus (TuMV) NIa [25], alleviated AD-associated symptoms in animal models. These findings lead us to hypothesize that proteolytic cleavage of polyQ stretches would be a valid approach for the treatment of polyQ diseases. Since no protease was known to cleave polyQ stretches, we decided to generate one using a combination of rational design and directed evolution approaches.

We started protein engineering with several viral proteases, including the 3C proteases (3CP) of mengovirus, coxsackievirus, and hepatitis A virus (HAV). One important feature shared by these proteases is that they cleave peptide bonds after glutamine. Our preliminary studies revealed that mengovirus and coxsackievirus 3CPs strictly favored a proline residue at their P2′ sites, while HAV 3CP virtually favored any amino acid at the P2′ position. Due to its leniency in substrate requirement, HAV 3CP was utilized for further engineering. The glutamine scanning experiments showed that the P2 and P1' sites did not accommodate glutamine residues, hence necessitating modifications in the S2 and S1′ pockets of HAV 3CP.

One of the main reasons for the low rate of success in protease engineering has been a lack of efficient and comprehensive screening procedures for exploring the cleavage site preferences of engineered proteases. In this study, the task of screening the variant library was made relatively easier by the use of the genetic screening method, GASP. GASP has been successfully used in the past in protease substrate specificity studies [16], [26], [27,]. The current study provides further evidence that this procedure is an efficient screening method for isolating protease variants with a desired substrate specificity from a pool of protease variants. Other methods that involve displaying the enzyme in a membrane to enable it to react with exogenous synthetic substrates have also been successfully exploited to isolate variants with a desired substrate selectivity [28]. However, GASP in particular has been proven to be effective for the study of cytosolic proteases. In this study, HAV 3CP variants were successfully isolated that can cleave polyQ stretches as assayed by GASP. One of the most active variants, Var26, prevented polyQ-induced cytotoxicity and thus confirmed our hypothesis that the cleavage of polyQ stretch is a valuable approach for the treatment of polyQ diseases. To ensure that Var26 was catalytically active and that the Var26-mediated prevention of polyQ-induced cytotoxicity was proteolytic activity-dependent, an active site mutant of Var26, C172A, was generated. Our results clearly show that C172A fails to rescue neuronal cells from polyQ-induced toxicity, which indicates that the beneficial effects of Var26 are proteolytic activity-dependent. Immunoblotting showed that only a small portion of the toxic HttEx1(97Q)-GFP protein is degraded by Var26 (Fig. 3C). Therefore, further study of the exact molecular mechanisms underlying the effects of Var26 is warranted.

We expected that the variant proteases would cleave anywhere within the polyQ region, thereby making the longer aggregation-prone polyQ stretches shorter and rendering them less susceptible to aggregation. In this study, the variant proteases were not directed specifically against longer polyQ stretches because our screening assay did not discriminate the length of the polyQ stretch. Therefore, it was quite surprising to see that the HttEx1(25Q)-GFP protein was not affected by the presence of Var26, while HttEx1(97Q) was substantially degraded (Fig. 3C). We presume that this could mainly be due to structural differences between the substrates. Because it has been previously shown that compared to shorter polyQ stretches the longer or pathological lengths of polyQ containing proteins undergo a length dependent structural transition from α-helix to a β-sheet rich forms even in their monomeric state prior to their assembly in to amyloid like fibrils. This structural transition was shown to be critical step in the cytotoxicity rather than the whole aggregation process itself [29], [30]. Therefore, the specificity of Var26 towards 97Q may arise from the structural transition. Also more likely the Var26 may attack the 97Q in its native monomeric state, a relatively more relaxed conformer of the long polyQ during their structural transition. The glutamine residues in the Q8 substrate expressed in yeast as a fusion protein and the polyQ stretch in HttEx1(97Q)-GFP may be more exposed relative to the glutamines in HttEx1(25Q)-GFP. Whatever the difference is, this unexpected capability of Var26 to discriminate between the substrates may open the way for the selective degradation of pathogenic polyQ stretch by a protease. While the proteolytic activity of Var26 and its beneficial effects in yeast cells and mammalian cultured cells were clearly observed, the polyQ cleaving activity of Var26 was not detected *in vitro* most likely due to its low catalytic activity (data not shown). This is understandable because some report claim that through directed evolution as many as 50 cycles of selection were needed to generate a protein with desired activity [31] compared to only two cycles of selection performed in this study. Overall, it appears that GASP is a sensitive assay and that beneficial effects can be obtained with a protease that has relatively low polyQ cleaving activity.

Much work remains to be done to explore the possibility of using a modified viral protease in gene therapy for HD. The potential for toxicity following the introduction of a viral protease in a disease model and the effectiveness of the protease in other polyQ disorders need to be studied. Nonetheless, this study provides a proof-of-concept for using a polyQ-specific protease (PQP) as a treatment for polyQ diseases. Besides broadening the potential use of GASP as a screening method in the field of protease engineering, this study opens-up a new window that provides a novel insight in the development of yet unexplored strategy to treat polyQ diseases.

## Materials and Methods

### Plasmid manipulation

For the yeast experiments, HAV 3CP and all of the engineered variants were cloned into the pGAL vector as described previously [16], and the construction of the substrate vector pADH-Ste2-Lex has been described elsewhere [32]. The various substrate linkers (listed in the Table S1) were cloned between the Nco1 and BamH1 sites of the substrate vector. For the mammalian cell culture work, HAV 3CP, the engineered variant 26, and the active site mutant of Var26 were cloned in to the EcoR1 and Xho1 sites of the pCDNA3 vector, resulting in pcDNA3-WT 3CP, pcDNA-Var26, and pcDNA3-C172A, respectively. The coding region of the Human Huntingtin exon1 with 25 or 97 glutamine repeats followed by an EGFP sequence was cloned between the Xho1 sites of pcDNA3, resulting in pcDNA-HttEx1(25Q) or (97Q)-GFP.

### Yeast transformation and screening

Yeast transformation and screening was performed as described by Sellamuthu et al [16].

### V28, M29, H145, and K146 randomization

Three sets of primers were used to make HAV 3CP in 3 fragments generated by successive PCR. Primer set STE-2-F and V28, M29 ran-R: 5′-TCC CAA GGC ATT MNN MNN CCA TCT CAC ACA-3′ was used to make fragment one. The primer set V28, M29 ran-F: 5′-TGT GTG AGA TGG NNK NNK AAT GCC TTG GGA-3′ and H145, K146 ran-R: 5′-ACC ATC ATT TTT MNN MNN AAC ATA AGT AGC-3′ was used to make fragment two. Primers H145, K146 ran-F: 5′-GCT ACT TAT GTT NNK NNK AAA AAT GAT GGT-3′ and HAV3C-R were used to generate fragment three. All of these three PCR products were used to make the full length V28, M29, H145 and K146 randomized HAV3C proteases. The library size was one million.

### M29, H145, K146, and K147 randomization

The S2 pocket residues comprising M29, H145, K146, and K147 were randomized using the external primers STE-2-F and HAV3C-R and two sets of randomized inner primers, (i) M29 ran-F, 5′-GTG AGA TGG GTT NNK AAT GCC TTG GGA-3′; (ii) M29 ran-R, 5′-TCC CAA GGC ATT MNN AAC CCA TCT CAC-3′; (iii) HKK ran-F, 5′-GCT ACT TAT GTT NNK NNK NNK AAT GAT GGT ACA ACA-3′; and (iv) HKK ran-R, 5′-TGT TGT ACC ATC ATT MNN MNN MNN AAC ATA AGT AGC TTT-3′. One million individual colonies were obtained.

### L168, P169, and L199 randomization

The S1′ pocket residues L168, P169, and L199 were randomized to generate a mutant protease library using the set of external primers HAV3C-F and ADH-ter-R, along with randomized inner primers, (i) L168, P169 ran-F: 5′-AAA GGC GAA GGT NNK NNK GGA ATG TGT GGT-3′; (ii) L168, P169 ran-R: 5′-ACC ACA CAT TCC MNN MNN ACC TTC GCC TTT-3′; (iii) L199 ran-F: 5′-GGA AAT TCA ATT NNK GTT GCA AAA TTG-3′; and (iv) L199 ran-R: 5′-CAA TTT TGC AAC MNN AAT TGA ATT TCC-3′. A library of 70,000 colonies was obtained.

### Combining the S2 and S1′ pocket mutants

Individually generated S2 and S1′ pocket mutations were combined to make the full length protease so that both S2 and S1′ pocket modifications were present in the same variant. This was performed by two-step PCR based shuffling. First, a part of the protease fragment containing the S2 pocket region was amplified by PCR from the entire P2-Q substrate cleaving, S2 pocket randomized library of variants. This included 12 candidates from the HKKL mutants, 23 candidates from the VMHK mutants, and 19 candidates from the MHKK library (see Tables S2 and S3). Next, a downstream fragment of the protease containing the S1′ region was PCR amplified from all of the 23 candidates of the P1'-Q substrate cleaving, S1' pocket randomized LPL library (see Table S4). These two individual PCR fragments, one which contained a S2 pocket modification and the other a S1′ modification, were combined and used as a template to generate the full length protease. The final PCR product composed the protease with modifications in both of the pockets.

### Directed evolution

Variant proteases with S2 and S1′ pocket mutations were subjected to two rounds of error prone PCR (epPCR) and shuffling. The epPCR was carried out using a Diversify PCR random mutagenesis kit from Clontech according to the manufacturer's instructions. The average mutation rate was 2∼3 mutations per 1000 base pairs. DNA shuffling was performed as described by Stemmer [33].

### Reagents and antibodies

Cell culture media and fetal bovine serum were purchased from Hyclone (Logan, UT, USA) and antibiotics were purchased from Invitrogen (Carlsbad, CA, USA). The antibodies used in this study were as follows: a rabbit polyclonal anti-LexA DNA binding region antibody (ab14553, Abcam, Cambridge, MA, UK); a mouse monoclonal anti-huntingtin antibody (MAB5374, Millipore, Billerica, MA, USA); a rabbit polyclonal anti-active Caspase-3 antibody (ab13847, Abcam); and a mouse monoclonal anti-α-tubulin antibody (sc-5286, Santa Cruz Biotechnology, Santa Cruz, CA, USA). The HP12 antibody recognizing the first 17 amino acids of Htt was kindly provided by Dr. Ihn Sik Seong (Harvard Medical School). Unless otherwise mentioned, all chemicals were purchased from Sigma.

### Cell culture and transient cotransfection

SH-SY5Y human neuroblastoma cells were grown in Dulbecco's modified Eagle medium supplemented with 10% fetal bovine serum and 100 U/100 µg/ml penicillin/streptomycin. The plasmids pcDNA3-WT 3CP, Var26, or C172A were cotransfected along with pcDNA3-HttEx1(25Q) or (97Q)-GFP into cells using Lipofectamine LTX (Invitrogen). As an empty vector control, a matching vector without an insert (pcDNA3, Invitrogen) was used. To ensure that all cells expressing HttEx1-GFP also expressed the appropriate protease, a 3∶1 ratio of protease DNA to HttEx1-GFP DNA was used.

### Immunoblotting

For yeast cell immunoblotting, EGY48 cells grown in selective drop out medium with either glucose or galactose/raffinose as a carbon source were harvested by centrifugation for 2 min at room temperature. Cells were resuspended in SUMEB buffer (1% SDS, 8 M Urea, 10 mM MOPS [pH 6.8], 10 mM EDTA, 0.01% Bromophenol blue) containing a protease inhibitor cocktail and were lysed by glass bead vortexing. After incubation for 10 min at 65°C, lysates were removed from the beads by centrifugation at 1,500 g for 5 min at room temperature. The supernatant was then subjected to SDS-PAGE (10%) and transferred to PVDF membranes (Bio-Rad Laboratories). Membranes were blocked with 5% non-fat dry milk powder in TBS-T for 1 hr. Incubation with the primary antibody was performed overnight at 4°C. After incubation with peroxidase-labeled anti-mouse or anti-rabbit antibodies (1∶10,000, Zymed Laboratories), the immune complexes were visualized using ECL reagent (Amersham Pharmacia).

For mammalian cell immunoblotting, SH-SY5Y cells were harvested and resuspended in lysis buffer (1% NP-40, 50 mM Tris-HCl [pH 7.4], 150 mM NaCl, 10 mM NaF) supplemented with a mammalian cell protease inhibitor cocktail and sonicated briefly. The soluble protein fraction was recovered after centrifugation at 10,000 g for 1 min and the supernatants were subjected to SDS-PAGE (12%). The protein concentration was determined using the BCA method. Protein loading was controlled by probing for α-tubulin or GAPDH on the same membrane and the intensity of each band was quantified by densitometry (Bio-Rad).

### Immunofluorescence

SH-SY5Y cells were grown on poly-L-lysine-coated coverslips, and after the experimental procedure, the cells were fixed with 4% paraformaldehyde (pH 7.4)/TBS for 10 min, washed twice, and permeabilized in 0.5% Triton X-100/TBS for 5 min at room temperature. After three washes with TBS containing 1 mM CaCl_2_ and 1 mM MgCl_2_, the cells were exposed to HP12 antibody for 30 min at 37°C or 1 hour at room temperature. The fixed cells were then rinsed in TBS, incubated with Alexa 594-conjugated secondary antibody (Invitrogen) for 30 min at 37°C or 1 hour at room temperature and mounted in PermaFlouor Aqueous Mountant (Lab vision Corporation). Fluorescence images were visualized using a Leica DMRBE microscope equipped with a 63x (1.4NA) oil objective and fluoresceine isothiocyanate- or Texas Red-optimized filter sets (Omega^R^ Optical Inc, Brattleboro, VT, USA). Images were acquired using a CoolSNAP ™fx CCD camera and analyzed with Metamorph imaging software (Universal Imaging Co, Downingtown, PA, USA). To determine the levels of polyglutamine aggregation among GFP positive cells, the number of aggregation positive cells vs the number of GFP positive cells was counted in 40 random fields per culture.

### Estimation of polyglutamine aggregation and cell death/survival

SH-SY5Y cells were transfected with pcDNA3-HttEx1(25Q or 97Q)-GFP together with pcDNA-WT 3CP, Var26, or C172A plasmids, and the number of dying cells was estimated by by trypan blue staining. Cells were also examined for apoptotic pyknotic nuclei by Hoechst staining following the methods described by Wyttenbach et al [22], and Ye et al [34].

## Supporting Information

Figure S1 Expression of Var26 is not cytotoxic. SH-SY5Y cells were transfected with pcDNA3-WT 3CP (WT), pcDNA3-Var26 (Var26), or pcDNA3-C172A (C172A) or pcDNA-HttEx1(97Q)-GFP. After 0, 24, 48 and 72 hours of incubation, the number of surviving cell population was assessed by MTT assay. The percentage of surviving cells was plotted against time. Error bars represent the SD. Significance was determined by student's t test. *p<0.05.
(EPS)

Table S1 Various linker sequences.
(DOCX)

Table S2 P2-Q substrate-cleaving variants selected from a library randomized at amino acids V28, M29, H145, and K146.
(DOCX)

Table S3 P2-Q substrate-cleaving variants selected from a library randomized at amino acids M29, H145, K146, and K147.
(DOCX)

Table S4 P1'-Q substrate-cleaving variants selected from a library randomized at amino acids L168, P169, and L199.
(DOCX)

Table S5 Q8 substrate-cleaving variants selected from a library containing combined mutations in the S2 and S1' pockets.
(DOCX)

Table S6 The variants selected in the final screens that show moderate proteolytic activity for the Q8 substrate.
(DOCX)
