# Supplementary material for: An Engineered Viral Protease Exhibiting Substrate Specificity for a Polyglutamine Stretch Prevents Polyglutamine-Induced Neuronal Cell Death
Source: PLoS One. 2011 Jul 20;6(7):e22554. doi: 10.1371/journal.pone.0022554 (PMC3140514; doi:10.1371/journal.pone.0022554)
Supplement: Table S1 — Various linker sequences. (DOCX) [file pone.0022554.s002.docx]

| Plasmid name | Substrate name | Substrate sequence | | | | | | | | | | | |
| --- | --- | --- | --- | --- | --- | --- | --- | --- | --- | --- | --- | --- | --- |
|  |  | P8 | P7 | P6 | P5 | P4 | P3 | P2 | P1 | P1' | P2' | P3' | P4' |
| pADH-Ste2-2B/2C-Lex | Wt | R | M | M | E | L | R | T | Q | S | F | S | N |
| pADH-Ste2-P2Q-Lex | P2-Q | R | M | M | E | L | R | Q | Q | S | F | S | N |
| pADH-Ste2-P3Q-Lex | P3-Q | R | M | M | E | L | Q | T | Q | S | F | S | N |
| pADH-Ste2-P4Q-Lex | P4-Q | R | M | M | E | Q | R | T | Q | S | F | S | N |
| pADH-Ste2-P5Q-Lex | P5-Q | R | M | M | Q | L | R | T | Q | S | F | S | N |
| pADH-Ste2-P1'Q-Lex | P1'-Q | R | M | M | E | L | R | T | Q | Q | F | S | N |
| pADH-Ste2-P2'Q-Lex | P2'-Q | R | M | M | E | L | R | T | Q | S | Q | S | N |
| pADH-Ste2-P3'Q-Lex | P3'-Q | R | M | M | E | L | R | T | Q | S | F | Q | N |
| pADH-Ste2-P5-3'Q-Lex | P5-3'Q(TS) | R | M | M | Q | Q | Q | T | Q | S | Q | Q | N |
| pADH-Ste2-Q8-Lex | Q8 | R | M | M | Q | Q | Q | Q | Q | Q | Q | Q | N |
