# Supplementary material for: An Engineered Viral Protease Exhibiting Substrate Specificity for a Polyglutamine Stretch Prevents Polyglutamine-Induced Neuronal Cell Death
Source: PLoS One. 2011 Jul 20;6(7):e22554. doi: 10.1371/journal.pone.0022554 (PMC3140514; doi:10.1371/journal.pone.0022554)
Supplement: Table S2 — P2-Q substrate-cleaving variants selected from a library randomized at amino acids V28, M29, H145, and K146. (DOCX) [file pone.0022554.s003.docx]

| **Sample**  **No.** | **V28** | **M29** | **H145** | **K146** |
| --- | --- | --- | --- | --- |
| 1 | A | C | G | S |
| 2 | A | C | A | A |
| 3 | A | C | G | Q |
| 4 | V | V | G | Q |
| 5 | V | V | G | E |
| 6 | V | V | G | A |
| 7 | V | V | G | T |
| 8 | V | C | G | L |
| 9 | V | C | G | S |
| 10 | V | C | G | A |
| 11 | V | C | G | C |
| 12 | V | C | G | V |
| 13 | V | G | L | G |
| 14 | C | L | G | E |
| 15 | C | C | G | L |
| 16 | C | C | C | E |
| 17 | R | S | G | D |
| 18 | R | C | G | Q |
| 19 | G | C | A | E |
| 20 | L | T | S | E |
| 21 | L | A | G | E |
| 22 | L | V | G | L |
| 23 | E | L | G | L |
