# Supplementary material for: An Engineered Viral Protease Exhibiting Substrate Specificity for a Polyglutamine Stretch Prevents Polyglutamine-Induced Neuronal Cell Death
Source: PLoS One. 2011 Jul 20;6(7):e22554. doi: 10.1371/journal.pone.0022554 (PMC3140514; doi:10.1371/journal.pone.0022554)
Supplement: Table S3 — P2-Q substrate-cleaving variants selected from a library randomized at amino acids M29, H145, K146, and K147. (DOCX) [file pone.0022554.s004.docx]

| **Sample No** | **M29** | **H145** | **K146** | **K147** |
| --- | --- | --- | --- | --- |
| 1 | V | G | C | S |
| 2 | V | G | E | G |
| 3 | L | G | E | G |
| 4 | L | G | E | K |
| 5 | V | G | V | G |
| 6 | V | G | V | D |
| 7 | V | G | E | A |
| 8 | T | G | E | A |
| 9 | T | G | E | S |
| 10 | T | G | E | R |
| 11 | V | G | E | R |
| 12 | V | G | Y | G |
| 13 | V | G | Q | S |
| 14 | V | G | V | Q |
| 15 | V | G | Q | R |
| 16 | V | G | Q | A |
| 17 | V | G | L | H |
| 18 | V | G | W | H |
| 19 | V | A | D | A |
