# Supplementary material for: An Engineered Viral Protease Exhibiting Substrate Specificity for a Polyglutamine Stretch Prevents Polyglutamine-Induced Neuronal Cell Death
Source: PLoS One. 2011 Jul 20;6(7):e22554. doi: 10.1371/journal.pone.0022554 (PMC3140514; doi:10.1371/journal.pone.0022554)
Supplement: Table S4 — P1'-Q substrate-cleaving variants selected from a library randomized at amino acids L168, P169, and L199. (DOCX) [file pone.0022554.s005.docx]

| **Sample no.** | **L168** | **P169** | **L199** |
| --- | --- | --- | --- |
| 1 | L | P | C |
| 2 | L | G | C |
| 3 | R | G | A |
| 4 | R | G | V |
| 5 | Q | G | V |
| 6 | Q | G | A |
| 7 | R | P | V |
| 8 | R | P | A |
| 9 | R | P | C |
| 10 | R | A | C |
| 11 | R | A | A |
| 12 | Q | P | V |
| 13 | R | E | C |
| 14 | R | S | C |
| 15 | V | G | V |
| 16 | V | G | C |
| 17 | V | P | C |
| 18 | A | P | C |
| 19 | M | P | C |
| 20 | K | P | M |
