# Supplementary material for: An Engineered Viral Protease Exhibiting Substrate Specificity for a Polyglutamine Stretch Prevents Polyglutamine-Induced Neuronal Cell Death
Source: PLoS One. 2011 Jul 20;6(7):e22554. doi: 10.1371/journal.pone.0022554 (PMC3140514; doi:10.1371/journal.pone.0022554)
Supplement: Table S5 — Q8 substrate-cleaving variants selected from a library containing combined mutations in the S2 and S1' pockets. (DOCX) [file pone.0022554.s006.docx]

| **Sample No** | **V28** | **M29** | **H145** | **K146** | **K147** | **L155** | **L168** | **P169** | **L199** |
| --- | --- | --- | --- | --- | --- | --- | --- | --- | --- |
| 1 | A | V | G | E | A | L | Q | P | C |
| 2 | V | V | G | E | A | L | R | A | C |
| 3 | V | V | G | E | A | L | K | P | C |
| 4 | V | V | G | E | A | L | R | P | C |
| 5 | V | V | G | E | K | L | R | P | C |
| 6 | V | V | G | E | K | I | R | P | C |
| 7 | V | V | G | E | D | L | A | P | C |
| 8 | Q | T | G | F | E | L | R | S | C |
| 9 | L | V | G | E | A | L | K | P | C |
| 10 | L | V | G | E | K | L | R | P | C |
| 11 | E | L | G | L | K | L | Q | P | C |
