# Supplementary material for: An Engineered Viral Protease Exhibiting Substrate Specificity for a Polyglutamine Stretch Prevents Polyglutamine-Induced Neuronal Cell Death
Source: PLoS One. 2011 Jul 20;6(7):e22554. doi: 10.1371/journal.pone.0022554 (PMC3140514; doi:10.1371/journal.pone.0022554)
Supplement: Table S6 — The variants selected in the final screens that show moderate proteolytic activity for the Q8 substrate. (DOCX) [file pone.0022554.s007.docx]

| **Clone** | **Mutations** |
| --- | --- |
| Var1 | V28A M29V H145G K146E K147A L168Q Q181L L199C |
| Var2 | V28A M29V Y65N K89R T126S H145G K146E K147D L168K L199C |
| Var3 | V28Q M29V H145G K146F K147E L168K L199C |
| Var4 | V28L M29V G62D I75T H145G K146E K147D D154E L168A L199C |
| Var5 | N22D V28A M29V R115C H145G K146E K147D L168K L199C |
| Var6 | V28A M29V E53K H145G K146E K147A L168K Q181R L199C |
| Var7 | V28A M29V V74A H145G K146F K147E L168Q L199C |
| Var8 | I5M V28Q M29T K35R H145G K146E K147D L168A L199C |
| Var9 | V28A M29V H145G K146F K147E L168Q L199C |
| Var10 | M29V H145G K146E K147A N148S L168R Q181R L199C |
| Var11 | V28A M29V D98N H145G K146F K147E L168R Q181L L199C |
| Var12 | M29V D37E H145G K146E L168R Q181L L199C |
| Var13 | V28A M29V D37E H145G K146E K147D D154N A160V L168K L199C |
| Var14 | V28Q M29T P94L N114S H145G K146F K147E L168R L199C |
| Var15 | G23V V28Q M29T K35R H145G K146E K147A L168K L199C |
| Var16 | V9I V28A M29V Q76R K95R A141T H145G K146E K147A L168Q L199C |
| Var17 | V28L M29V M54K H145G K146E K147D L168A L199C |
| Var18 | M29V H145G K146E K147D K164R L168A L199C |
| Var19 | V28A M29V V74A H145G K146E K147A L168K L199C |
| Var20 | V28L M29V V74D M137T H145G K146E L168R Q181L L199C |
| Var21 | V14A V28T M29V S131P H145G K146E L168Q Q181L L199C |
| Var22 | M29V H145G K146E K147D T151I D154E L168A L199C |
| Var23 | V28Q M29T K35R H145G K146E K147D L168A L199C |
| Var24 | V28A M29V T122A H145G K146E K147D L168R Q181R L199C |
| Var25 | M29V T118I H145G K146E K147A L168R L199C |
| Var26 | V28A M29V H145G K146F K147E L168K Q181L L199C |
| Var27 | V28A M29V V80E H145G K146E K164R L168K L199C |
| Var28 | V28L M29V Y66C K89R L116P H145G K146E K147A 168K L199C |
| Var29 | V28L M29V N114D H145G K146E L168R Q181L L199C |
| Var30 | V28Q M29T P134S H145G K146E K147D D154E L168K Q181R L199C F209L |
| Var31 | M29V H145G K146E K147D D154E L168A L199C |
| Var32 | V28A M29V H145G K146F K147E L168K L199C |
| Var33 | V28L M29V V90I H145G K146E K147A K164R L168K L199C |
| Var34 | M29V D36G S131P H145G K146E K147A K164I L168R Q181R L199C |
| Var35 | V28A M29V D36E I93M H145G K146E K147A L168Q L199C |
| Var36 | M29V E138K H145G K146E K147A L168K Q181R L199C |
| Var37 | V28A M29V I75T K89R P110S H145G K146E K147D D154E L168A L199C |
| Var38 | V28L M29V H145G K146E L168R Q181L S197P L199C |
| Var39 | V28L M29V D36G D37E R61I G71S V85A H145G K146E L168Q L199C |
| Var40 | V28A M29V S67T D108N H145G K146F K147E L168R L199C |
| Var41 | V28Q M29T S131T H145G K146E K147D L168A L199C |
| Var42 | V18I V28A M29V H145G K146E K147A L168Q Q181R L199C K215E |
| Var43 | V28L M29V I93F H145G K146E K147D L168K L199C Q210R |
| Var44 | V28Q M29T K35R N72T H145G K146E K147N L168A Q181R L199C |
